# Pd(0)-Mediated Deallylation Chemistry: A Reassessment of Its Application in Sensing CO

**DOI:** 10.1021/acs.joc.5c01628

**Published:** 2025-11-04

**Authors:** Dongning Liu, Xiaoxiao Yang, Shivanagababu Challa, Hongliang Li, Binghe Wang

**Affiliations:** Department of Chemistry and Center for Diagnostics and Therapeutics, 1373 Georgia State University, Atlanta, Georgia 30303, United States

## Abstract

Pd­(0)-mediated deallylation has been employed for developing fluorescent probes for carbon monoxide (CO). The key idea relied on the ability of CO to reduce Pd­(II) to Pd(0). However, most studies used Ru-based CORM-2 and/or CORM-3 as CO sources, despite their known chemical reactivity and idiosyncratic CO release. Herein, we conducted studies using one of the most widely used probes (**FL-CO-1**), evaluating its response to various CO sources and to Pd(0). We found that (1) the activation of **FL-CO-1** by CORM-2/-3 has CO-independent component(s); (2) vitamin C and cysteine were found to interfere with the probe’s performance; and (3) Pd(0) only led to moderate fluorescence turn-on, while a combination of Pd(0) and CO resulted in a pronounced fluorescence turn-on response. Such findings indicate that the role(s) of CO goes beyond Pd­(II) reduction, and accurate *in vivo* detection of CO using this approach is unlikely because of the presence of vitamin C and thiols in living systems. These new insights suggest the need to reinterpret some results, particularly when chemically reactive CORM-2 and CORM-3 were employed as CO surrogates. We recommend that future studies avoid using reactive CORMs to ensure experimental rigor.

## Graphical abstract

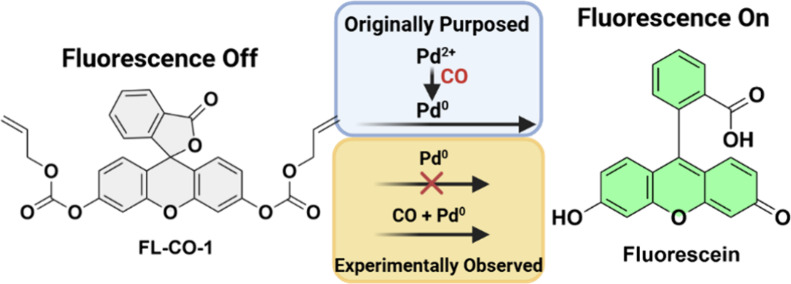

## Introduction

Palladium-mediated deallylation chemistry is well-established and has been applied in protein activation in live cells.^1,2^ This reaction requires Pd(0) as a catalyst.^3^ Notably, carbon monoxide (CO) has been reported to reduce Pd­(II) to Pd(0) under ambient conditions.^4^ A large number of publications (over 50) have described fluorescent probes for CO based on CO’s ability to reduce Pd­(II) to Pd(0) for the purpose of Pd(0)-mediated deallylation for fluorophore activation.^5,6^ In such systems, a stoichiometric amount of CO is required to reduce Pd^2+^ (e.g., PdCl_2_) to Pd(0), which was proposed to subsequently trigger deallylation and fluorescence activation (Scheme 1).

**Scheme 1:**
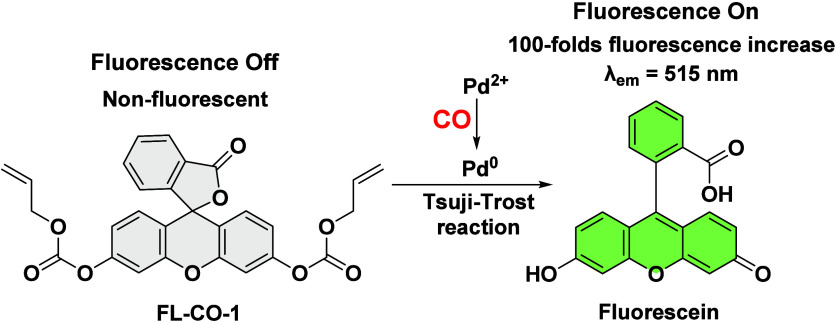
Originally Proposed Detection Mechanism of **FL-CO-1**

CO probes based on this mechanism employ diverse fluorophores such as fluorescein, rhodamine, nitrobenzofurazan, and boron-dipyrromethene, among others.^7^ Numerous solution-phase CO detection studies have been reported, often yielding impressive and visually striking results. We were intrigued by this detection method for three reasons. First, the rigor of the studies in most publications was established with the use of two Ru-based carbonyl complexes, CORM-2 and CORM-3, as the CO surrogates. These “CO donors” have also been used in hundreds of other publications.^8^ Unfortunately, recent studies have demonstrated a number of serious issues with the use of these CORMs as CO donors, including their extensive chemical reactivity,^9,10^ lack of CO production^11^ in the absence of a nucleophile or a redox agent, the predominant production of CO_2_ in aqueous solution,^12^ and nearly intractable CO production profiles when used in a cell culture or animal model studies,^8^ leading to the question of how to interpret the data of “CO sensing” using these probes that were only validated with chemically reactive CORMs. Therefore, there is an urgent need to re-examine the chemistry issues of these CORMs in the context of developing fluorescent probes for CO based on deallylation chemistry. Moreover, some recent publications on CO probes continue to employ these CORMs as CO surrogates, despite the well-documented issues associated with CORM-2 and CORM-3. This ongoing practice underscores the current relevance and importance of clarifying the limitations of such compounds in CO-sensing studies. Second, **FL-CO-1**–Pd­(II)–CO is a 3-component detection system with two sequential reactions. Therefore, there is an added complexity in terms of the reaction and detection kinetics. Third, unique to this detection system is the fact that Pd(0) is insoluble in an aqueous solution. Therefore, reduction of Pd­(II) by CO likely leads to the precipitation of Pd(0), which may present a colocalization issue for detection purposes, especially when used in cell culture or animal models. For all these reasons, we decided to dissect the chemistry by (1) comparing the effects of using different sources of CO; (2) studying the activation of the CO probe by using Pd(0), essentially bypassing the step of CO reduction of Pd­(II) and allowing us to focus on the second step of the fluorophore activation mechanism, the deallylation; and (3) examining whether commonly seen reducing agents in living systems, such as ascorbate or vitamin C, could pose an interference issue in bioanalysis. In doing so, we used CO probe **FL-CO-1** as an example because this is among the very first and most widely used probes in this category.^5^ The results obtained are striking and reveal a complex scenario, indicating that the data derived from the use of CORM-2 and CORM-3 do not represent the CO detection in any quantitative sense. Below, we discuss a detailed study.

## Results and Discussion

**FL-CO-1** was synthesized by following literature procedures^5^ and was fully characterized using NMR and MS to establish its identity (Figures S1–S3).

We first conducted experiments to examine the fluorescence turn-on deallylation reaction under different conditions using fluorescein at an equal molar concentration as a positive control. First, we should note that our findings using CORM-2 and CORM-3 are in general agreement with that described in the original publication,^5^ serving as secondary validation of the original results in this regard. However, there are some very interesting additional experimental findings that will help in the (re)­interpretation of results from similar deallylation-based probes.

When the time-dependent fluorescence turn-on of **FL-CO-1** (5 μM) was examined, both CORM-2 and CO solutions triggered time- and concentration-dependent fluorescence changes, as one would expect. However, very different time profiles between CORM-2 and CO in solution were observed (Figure 1). Most of the CO-solution experiments had a high initial value at zero time point (immediately after mixing) compared with the CORM-2 group. The CORM-2 experiments, on the other hand, showed that fast fluorescence intensity increases and plateaued off at about the 5 min point, at about 50% of the intensity of the positive control (about 600 at 5 μM fluorescein, Figure 2B­(a)). The results from the CORM-3 experiments were similar (see Figures 2A and 3A). In contrast, the fluorescence intensity of the CO-solution experiments showed a steady increase in the duration of the experiments (∼40 min). The different time profile kinetics mean that the difference in fluorescence intensity readings for these two types of experiments are time-dependent, even if they have the same amount of CO. If we take a snapshot of the results at the 30 min time point, this point becomes very clear. We should note that at 30 min, the CORM-2 experiments had already long-reached the plateau point and are no longer time-sensitive. However, the CO-solution experiments had not reached either the plateau point or completion of the reaction (i.e., total consumption of the fluorescent probe, **FL-CO-1**). It is clear in Figure 2A that the fluorescence intensity of the CO-solution experiments at 750 μM is about the same as that of CORM-2/CORM-3 at a much lower concentration (100 μM), while the fluorescence intensity from the experiments using a CO solution at 100 μM (Figure 2A) was lower than that of CORM-2/CORM-3 at the same concentration. However, at the 5 min time point, 100 μM of CORM-2/CORM-3 gave a higher fluorescence level than the CO-solution groups at either 100 or 750 μM. Such results suggest the CO-independent effects of CORM-2/CORM-3 and a lack of correlation of the CORM-2/CORM-3 experiments with that of pure CO in solution.

**Figure 1:**
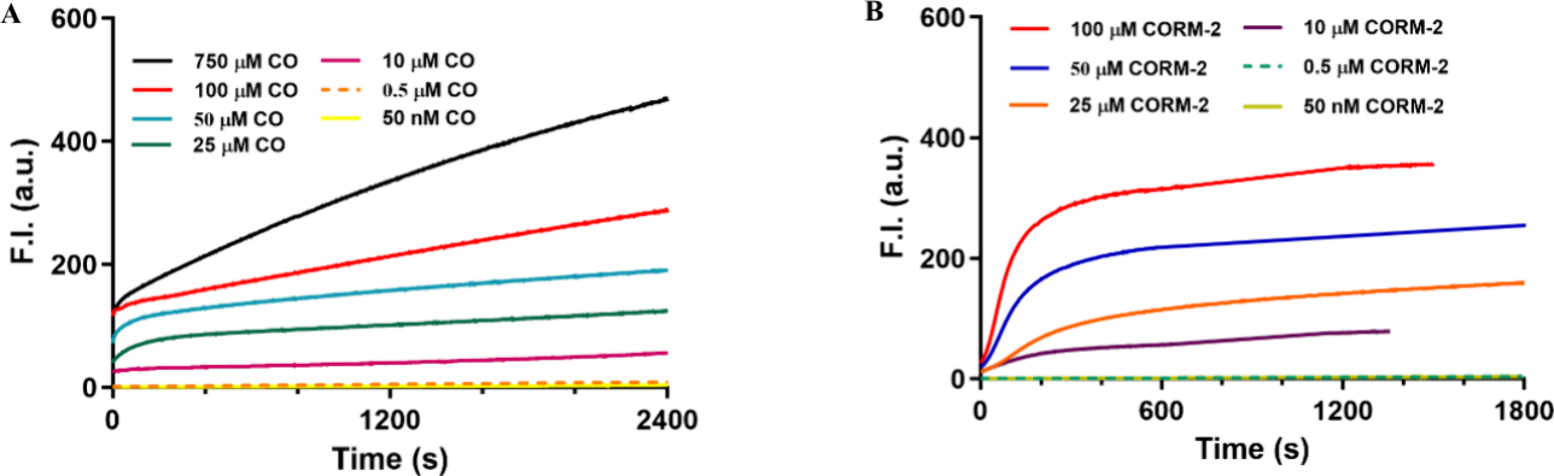
Fluorescence time-course **FL-CO-1** (5 μM) + PdCl_2_ (1 equiv) system in the presence of different concentrations of CO gas (A) in a mixed solution of PBS (99.8%), DMSO (0.2%), and CORM-2 (B) in a mixed solution of PBS (99%), DMSO (0.2%), and DMA (0.8%) at room temperature. The cuvette was sealed with a cap and mixed by vortexing for 5 s and then kept still for measurement (bandwidth = 3 nm, λ_ex_ = 490 nm, λ_em_ = 515 nm).

**Figure 2:**
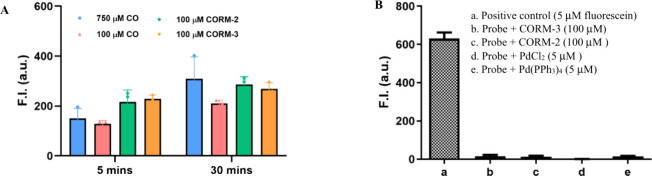
(A) Fluorescence intensity of **FL-CO-1** (5 μM) and PdCl_2_ (5 μM) in the presence of different concentrations of CO or CORM-2/CORM-3 after incubation for 5 or 30 min. (B) Fluorescence intensity of **FL-CO-1** (5 μM) after 30 min of incubation with different reactants at room temperature. **FL-CO-1**, PdCl_2_, and Pd­(PPh_3_)_4_ were prepared as 5 mM stock solution in DMSO; CORM-3 was prepared as 10 mM stock solution in deionized water, CORM-2 was prepared as 10 mM stock solution in DMA; 5 μM fluorescein was used as a positive control. Saturated CO solution was made by bubbling CO gas for 30 min. The experiments were carried out in a mixed solution of PBS (99%), DMSO (0.2%), and DMA (0.8%) at room temperature. The cuvette was sealed with a cap and mixed by vortexing for 5 s and then kept still for measurement (*n* = 3, mean ± SD, bandwidth = 3 nm, λ_ex_ = 490 nm, λ_em_ = 515 nm).

**Figure 3:**
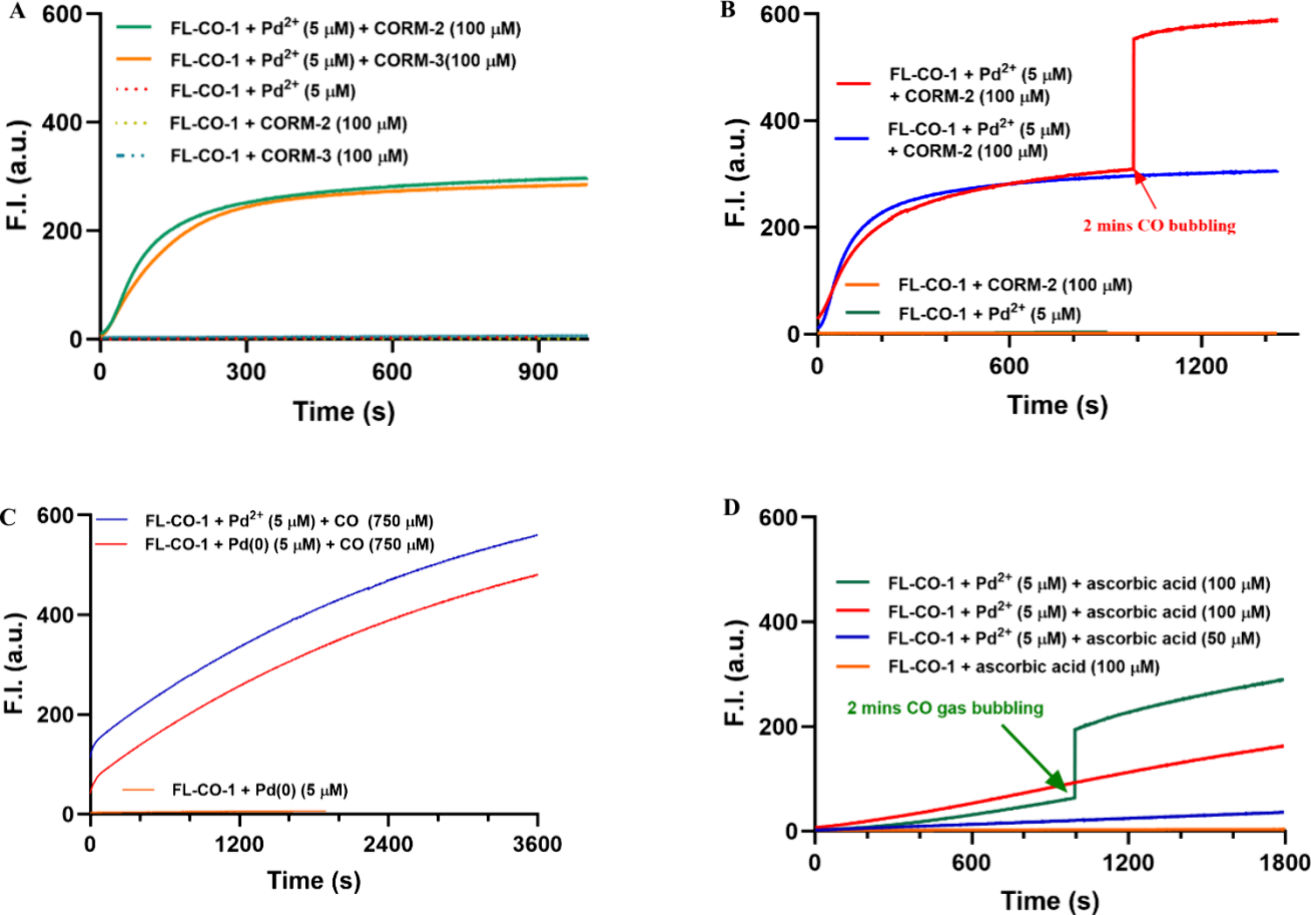
Time-dependent fluorescence profiles of **FL-CO-1** (5 μM) in different reaction systems at room temperature. (A) **FL-CO-1** with CORM-2 or CORM-3 in the presence of PdCl_2_. (B) **FL-CO-1** with CORM-2 or CORM-3 in the presence of PdCl_2_, with/without CO gas bubbling for 2 min. (C) **FL-CO-1** with PdCl_2_ (5 μM) or Pd(0) (5 μM) in a CO solution. (D) **FL-CO-1** with ascorbic acid in the presence of PdCl_2_, with/without CO gas bubbling for 2 min. The experiments were carried out in a mixed solution of PBS (99%), DMSO (0.2%), and DMA (0.8%) at room temperature. The cuvette was sealed with a cap and mixed by vortexing for 5 s and then kept still for measurement (bandwidth = 3 nm, λ_ex_ = 490 nm, λ_em_ = 515 nm).

Because the proposed mechanism of fluorescence turn-on was based on the ability of CO to reduce Pd­(II) to Pd(0) for catalysis of a deallylation reaction, we thought of using Pd(0) directly and thus bypassing the reduction step as a way to gain additional understanding of the sensing reaction. Much to our surprise, incubation with 5 μM of Pd(0) did not lead to a significant fluorescence turn-on (Figure 2B­(e)), with the fluorescence intensity of the solution being slightly above the negative control using Pd­(II) (Figure 2B­(d)) and about the same as CORM-2/CORM-3 alone without Pd­(II) (Figure 2B­(b,c)), which is clearly above the background (Figure S6B). The fluorescence turn-on effect of Pd(0) is far lower than that of the CORM-2/CORM-3-Pd­(II) or Pd­(II)-CO-solution experiments (Figure 2A and 3C). This was very surprising because in the originally proposed mechanism, the formation/availability of Pd(0) was proposed to be both essential and sufficient to catalyze the deallylation reaction, leading to fluorescence turn-on. The results from the Pd(0) experiments suggest the need to consider other factors beyond the CO-mediated reduction of Pd­(II) to Pd(0) as the roles of CO. This point is discussed later based on the additional experimental findings.

To further understand the chemistry behind the observations, we conducted additional experiments on the time-dependent fluorescence changes under various conditions. Fluorescence intensity increased rapidly for the first 250 s in the experiments with CORM-2/CORM-3 and then plateaued off quickly at about 50% conversion level (Figures 2A and 3A), which is similar to the results from many published deallylation-based probes as summarized in a recent review.^7^ In contrast, control experiments using either CORM-2/CORM-3 or Pd­(II) alone only led to very small amounts (Figure S6B) or no fluorescence intensity changes, respectively (Figure 2A). The fact that the fluorescence intensity plateaus off at 50% conversion indicates possibly slow capturing of the CO release from either CORM-2 or CORM-3 because the maximal amount of CO from each is 40 and 60 μM, respectively. Such numbers are based on prior literature studies, indicating that the maximal number of CO molecules that CORM-3 can release (3 molecules of CO per CORM-3) is higher than that of CORM-2 (1–2 CO molecules).^8^ To analyze the reasons for the 50% conversion yield, one can think of catalytic efficiency and turnover number of Pd(0) as a limiting factor or there may be other reasons. First, literature studies of similar deallylation reactions often used less than 5 mol % of the Pd(0) catalyst.^13,14^ However, in the current experiments, **FL-CO-1** and PdCl_2_ are used in a stoichiometric amount at 5 μM each. Therefore, the reduction of 5 mol % of PdCl_2_ to Pd(0) should suffice to catalyze the reaction to the near completion. However, that was obviously not the case. Therefore, we examined the next logical factor by bubbling CO (2 min) into the CORM-2 experiment/solution after it had reached the plateau phase. An additional quick enhancement in fluorescence intensity was observed (Figure 3B), reaching approximately 600, which is about the same as that of the positive control using 5 μM fluorescein (Figure 2B). Such results suggest that there was a sufficient amount of catalyst, presumably Pd(0), in the solution during the plateau phase, and near stoichiometric conversion of **FL-CO-1** to fluorescein was achievable when additional CO was available. The same experiments were conducted for CORM-3 (Figure S4A), showing a similar fluorescence enhancement upon CO bubbling. However, the magnitude of fluorescence intensity increase was lower, reaching only around 400 after CO gas bubbling. Such results again suggest a role of CO in the deallylation step, beyond the simple reduction of Pd­(II) to Pd(0). We made attempts to characterize the product(s) from reactions in the presence of CO without success. Such efforts were included to additional allyl ester analogs. It should be noted that the observed effects of CO in enhancing deallylation are consistent with the known role of CO in carbonylative coupling, carbonylative allyl transfer,^15^ and in coordinating to Pd(0).^16–18^ Incidentally, a new paper devoted its entire study to how chloride concentrations make a difference to Tsuji–Trost reaction.^19^ All these reports point to interesting but complex mechanistic questions. We figured that there is probably much more to study on the effects of additional factors, such as CO in combination with other components in the reaction mixture. For the sake of staying on the topic of understanding factors that could affect the performance of this deallylation-based CO probe, we did not further pursue the embedded organic chemistry question. The key message is that the role of CO goes beyond the reduction of Pd­(II) to Pd(0).

It is known that Pd­(II) reduction to Pd(0) leads to particle formation, which may lead to precipitation and/or aggregation and thus decreased catalytic efficiency.^4,20^ Therefore, we also wanted to include studies of agitation-mixing as an influencing factor. Consequently, we also tested vortex mixing and N_2_ bubbling (Figure S4B). While both N_2_ bubbling and vortexing caused a small increase in the fluorescence due to physical agitation, the enhancement observed with CO gas was unmistakenly due to added CO. Such results also indicate potential issues in Pd(0) precipitation or compartmentalization, when this detection system is used in cell culture or animal models.

Our experimental findings of the inability for Pd(0) (5 μM) to substantially turn on the fluorescence of **FL-CO-1** was surprising (Figure 2B­(e)). In terms of the reaction mechanism, Pd(0) is thought to be catalytic. However, when we increased the amount of Pd(0) from 5 to 100 μM, the fluorescence turn-on rate was substantially increased (Figure S5A). Very interestingly, adding CO to the Pd(0) reaction led to a faster reaction and very significant fluorescence intensity increase (Figure 3C), which again indicates a role for CO beyond Pd­(II) reduction in the activation of **FL-CO-1**. As such, CO seems to function as a reagent in at least two steps: Pd­(II) reduction and catalytic deallylation. It is also very interesting to note that the Pd­(II)–CO combination was moderately more effective at turning on the fluorescence of **FL-CO-1** than the Pd(0)–CO combination (Figure 3C), even though Pd(0) is proposed to be the catalytically active species. There are very interesting but complex chemistry questions involved.

Table 1 also summarizes the apparent *t*_1/2_ of the various reactions. Looking at these values, it becomes even clearer that reactions with CORM-2/-3 are much faster than those using CO gas or CO in solutions. Furthermore, the formation of Pd(0) does not correlate with CO detection, at least not quantitatively. The reactions with CORM-2 and CORM-3 are also much faster than using Pd(0) itself, although the originally proposed mechanism was said to be CO reduction of Pd­(II) to Pd(0) for the proposed catalytic deallylation reaction. All of these further raise many complex mechanistic questions, which are way beyond the scope of this manuscript. For example, ruthenium is a redox-active transition metal existing in multiple oxidation states, most notably +2, +3, and +4.^21^ Its rich coordination chemistry enables the formation of stable complexes with a variety of ligands, including phosphines, carbonyls, and π-accepting arenes.^21^ In organic synthesis, ruthenium-based catalysts play central roles in diverse transformations such as deallylation,^22,23^ including in aqueous solution,^22^ olefin metathesis, transfer hydrogenation, and C–H bond activation.^24^ Furthermore, CORM-2 and CORM-3 have been found to be redox active and catalytically active.^8–10,25^ It should also be noted again that CORM-2 and CORM-3 release CO_2_, not CO unless in the presence of a nucleophile or a redox-active agent.^8^ As such, results from CORM-2 and CORM-3 are unlikely a reflection of CO detection with Pd(0) as the catalyst, at least not entirely. For all of these reasons, it is not surprising that CORM-2 and CORM-3 behave differently from CO and Pd(0). Therefore, CORM-2 and CORM-3 have CO-independent effects and are not reliable CO donors for studying CO biology or chemistry.

**Table 1:** Reaction Half-Life and the Turn-On Percentage (Plateau Value Compared with Positive Control) of **FL-CO-1** (5 μM) with Different Components^a^

entry #	components	reaction half-life (s)	turn-on percentage of the positive control (%)
1	**FL-CO-1** + Pd^2+^ (5 μM) + CORM-3 (100 μM)	64 ± 6 (s)	45 ± 4%
2	**FL-CO-1** + Pd^2+^ (5 μM) + CORM-2 (100 μM)	46 ± 8 (s)	40 ± 1%
3	**FL-CO-1** + Pd^2+^ (5 μM) + CO gas (750 μM)	1784 ± 570 (s)	85 ± 8%
4	**FL-CO-1** + Pd0 (5 μM) + CO gas (750 μM)	2394 ± 199 (s)	94 ± 4%
5	**FL-CO-1** + Pd0 (100 μM)	762 ± 104 (s)	57 ± 5%

a *n* = 3, mean ± SD.

Along the line of studying applications of deallylation-based CO probes, we were interested in examining whether a reducing agent present in biological systems could pose interference problems, especially in studying CO concentrations and CO biology in cell culture and in animal models. Vitamin C or ascorbate is widely present in animals. For example, the mean concentration of vitamin C in human blood has been reported to be 8 mg/L (∼45 μM), with a range of 5–15 mg/L (∼28–85 μM).^26^ Therefore, we were interested in the effect of vitamin C on the performance of such CO detection systems. We studied the effects at two concentrations of vitamin C, 50 and 100 μM, and found very pronounced effects on fluorescence turn-on of the **FL-CO-1–**Pd­(II) system (Figure 3D). Similar to the previous studies (Figure 3B), the bubbling of the CO gas led to a quick further increase of the fluorescence intensity of the **FL-CO-1**–**** Pd­(II)–vitamin C system. Because vitamin C concentration can vary widely in the blood (∼28–85 μM), such results suggest intractable situations when this method is used for *in vivo* detection of CO under pathophysiological relevant conditions. We also studied the effect of l-cysteine, which exists in high abundance in cells.^27,28^ In the presence of l-cysteine, the fluorescence intensity increase for the system was significantly diminished (Figure S8B). Such results indicate interference by thiol species.^29–31^ Incidentally, during the revision process of this manuscript, the work by Manheri’s group indicated interference by thiol in this type of deallylation-based CO sensing.^31^

Finally, we also assess the detection limit of such a deallylation-based CO probe. First, it should be noted that the known concentration of free CO is low: ∼2 nM in human tissue and 2–10 pmol/mg in mouse organs.^32^ While the concentration of hemoglobin-bound CO, COHb, may exist at a high micromolar range under physiological conditions,^33^ Hb has a high affinity for CO: *K*_d_ of 0.7–2.7 nM in the high-affinity R-state and 4.5 μM in the low-affinity T-state,^33^ largely in peripheral tissue. Therefore, the free CO concentration in biological fluids is expected to be low. Furthermore, only the free CO fraction is relevant to reaction kinetic considerations. Given the need in earlier experiments to use much higher concentrations of CO or CORMs (high micromolar concentration) than what one can reasonably encounter under pathophysiological conditions, the question of the detection limit is an important one. For this study, we chose CORM-2 as an example of CORMs. Therefore, the reaction time profiles for **FL-CO-1** with dissolved CO and CORM-2 at different concentrations were examined (Figures 1, 4, and S6A). As shown in Figure 1, 10–100 μM of the CO gas led to a significant increase of fluorescence to the system after 40 min. A zoomed-in figure (Figure 4A) shows that 50 nM CO gas led to only marginal fluorescence increase compared with the control group. Similar results of CORM-2 are shown in Figure 4B. Considering the experimental errors of similar experiments, as shown in Figure 2, such readings do not allow for meaningful detection of CO at mid-nM concentrations. Furthermore, due to the low concentration of free CO in the biology system (∼2 nM in human tissue and 2–10 pmol/mg in mouse organs), it is hard to see how such a system can be reliably applied in determining physiologically relevant CO concentrations *in vivo*. This point becomes even more compelling when considering the strong fluorescence response induced by vitamin C—approximately 30% of the full turn-on signal (Figure 3D)—and the fact that vitamin C is present at relatively high concentrations (mid-micromolar range) in living systems.

**Figure 4:**
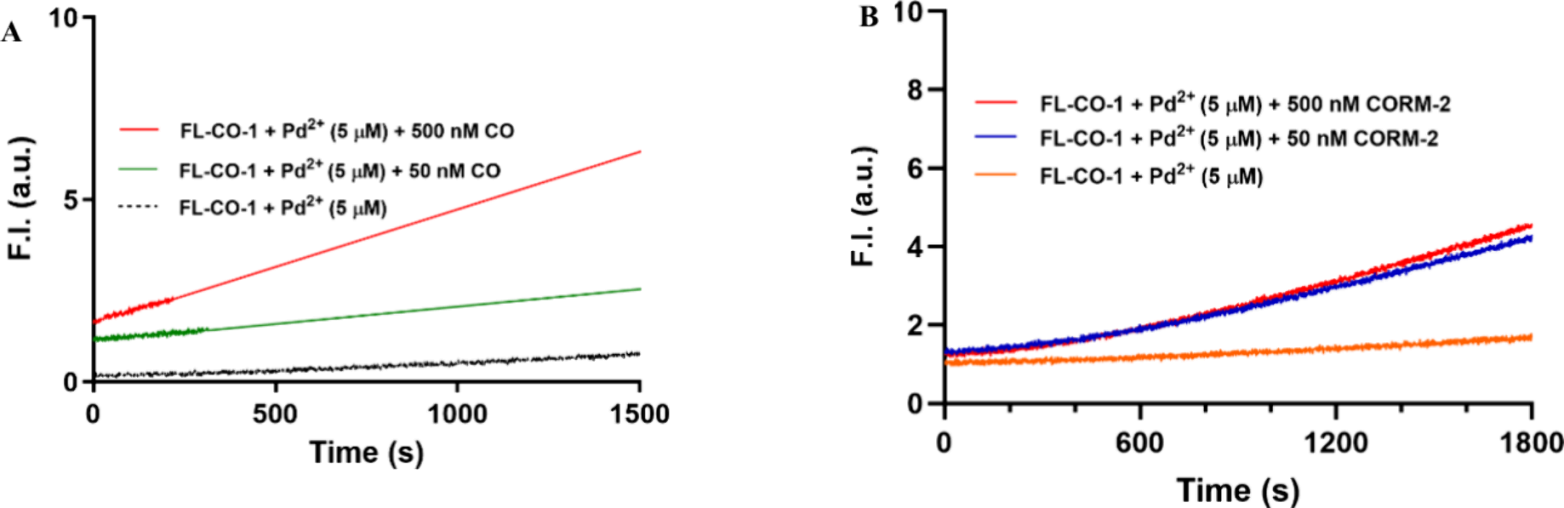
Fluorescence time-course comparison of CO gas and CORM-2 in the presence of the **FL-CO-1** (5 μM) + PdCl_2_ (5 μM) systems. (A) Time-dependent fluorescence intensity changes of **FL-CO-1** in the presence of Pd­(II) and dissolved CO at 500, 50 nM, and zero concentrations. (B) Time-dependent fluorescence intensity changes of **FL-CO-1** in the presence of Pd­(II) and CORM-2 at 500, 50 nM, and zero concentrations. The experiments were carried out in a mixed solution of PBS (99%), DMSO (0.2%), and DMA (0.8%) at room temperature. The cuvette was sealed with a cap and mixed by vortexing for 5 s and then kept still for measurement (bandwidth = 3 nm, λ_ex_ = 490 nm, λ_em_ = 515 nm).

## Conclusion

In this study, we systematically investigated the performance of a widely used deallylation-based CO probe, **FL-CO-1**, in the presence of Pd­(II) and various CO sources, including CORM-2, CORM-3, and pure CO gas under biologically relevant conditions. We also investigated the use of Pd(0) with and without CO. Our findings highlight four factors that influence the CO-sensing performance of deallylation-based CO probes. First, both CORM-2 and CORM-3 can activate the probes in a CO-independent mechanism, making them poor CO donors for such studies as have been demonstrated earlier.^8^ Second, Pd(0) alone did not cause a meaningful fluorescence turn-on, challenging the assumption that CO merely functions to reduce Pd­(II) to Pd(0) for probe activation. Third, the combination of Pd(0) and CO resulted in robust fluorescence activation, indicating a role for CO in the deallylation step. Fourth, certain biologically relevant agents, such as vitamin C and thiol, were also found to affect the performance of the **FL-CO-1**–Pd­(II) system, indicating issues with potential background signals and the probe’s selectivity under physiological conditions.

Collectively, these findings suggest the need to avoid using chemically reactive CORM-2 and CORM-3 as CO surrogates, the multiple roles that CO plays in activating the probe studied, and the need to consider interference issues with other biomolecules, such as vitamin C and thiol.

## Supplementary Material



## Data Availability

The data underlying this study are available in the published article and its Supporting Information.

## Notes

Material and instruments, experimental procedure, supporting figures, and NMR data (PDF)
