# Supplementary material for: Pd(0)-Mediated Deallylation Chemistry: A Reassessment of Its Application in Sensing CO
Source: J Org Chem. 2025 Nov 4;90(45):15965–71. doi: 10.1021/acs.joc.5c01628 (PMC12624833; doi:10.1021/acs.joc.5c01628)
Supplement: Supplementary file 1 [file jo5c01628_si_001.pdf]

Supplemental Information

Pd(0)-Mediated De-allylation Chemistry: A Re-assessment of Its Application in Sensing CO

Dongning Liu, Xiaoxiao Yang, Shivanagababu Challa, Hongliang Li, Binghe Wang*

Department of Chemistry and Center for Diagnostics and Therapeutics, Georgia State University, Atlanta, Georgia 30303 USA. Email: bwang31@gsu.edu.

Content

Experimental Section **S2**
Supporting Figures **S4**
References **S5**

Experimental Section

Material and Instruments

Chemical reagents and CORMs were purchased from Sigma-Aldrich (Saint Louis, MO) and/or Oakwood (Estill, SC). Solvents were purchased from Fisher Scientific (Pittsburgh, PA); and dry solvents were prepared using a Vigor Tech purification system (Houston, TX). Certified pure CO calibration gas was purchased from GASCO (Oldsmar, FL). Fluorescence spectra were recorded on a Shimadzu RF5301PC fluorometer (Kyoto, Japan). ^{1}H NMR (400 MHz) and ^{13}C NMR (101 MHz) were acquired on a Bruker AV-400 MHz Ultra Shield NMR.

Synthesis of the FL-CO-1

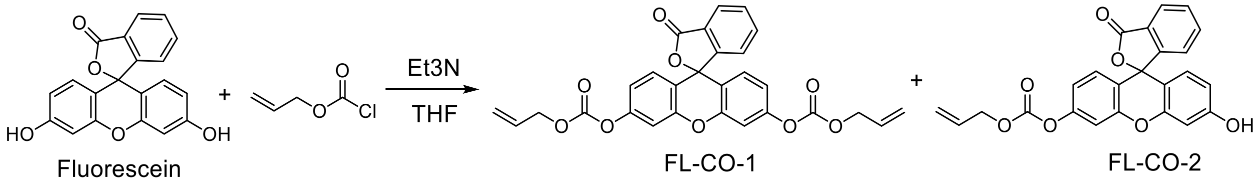

Scheme S1. Synthesis route of FL-CO-1.

FL-CO-1 was synthesized following a literature procedure (Scheme S1).[1] Briefly, under nitrogen, fluorescein (325 mg, 1.0 mmol) was dissolved in dry THF (10 mL), followed by Et_3N addition (417 µL, 3.0 mmol). After stirring for 10 min at room temperature, the reaction mixture was cooled to 0 ºC on ice bath before slow addition of allyl chloroformate (425 µL, 4 mmol) over 10 min. The resulting yellow mixture was then stirred overnight. After filtration, the filtrate was concentrated under reduced pressure to obtain the crude product, which was subsequently purified by column chromatography to obtain products **FL-CO-1** (white solid, 175 mg, 35 %) and **FL-CO-2** (light yellow sticky solid, 80 mg, 19 %). **FL-CO-1 :** (melting point: 77.1 ℃ - 80.2 ℃) ^{1}H NMR (400 MHz, $CDCl_3$): δ 8.04 (d, *J* = 7.3 Hz, 1H), 7.66 (dtd, *J* = 19.5, 7.3, 1.0 Hz, 2H), 7.18 (dd, *J* = 10.6, 4.8 Hz, 3H), 6.91 (dd, *J* = 8.7, 2.3 Hz, 2H), 6.84 (d, *J* = 8.7 Hz, 2H), 5.99 (ddt, *J* = 16.4, 10.5, 5.9 Hz, 2H), 5.44 (dd, *J* = 17.2, 1.3 Hz, 2H), 5.35 (dd, *J* = 10.4, 1.0 Hz, 2H), 4.75 (d, *J* = 5.9 Hz, 4H). ^{13}C {^{1}H} NMR (100 MHz, $CDCl_3$): δ 152.9, 152.4, 151.6, 135.5, 130.9, 130.2, 129.2, 125.4, 124.1, 120.0, 116.8, 110.0, 81.6, 69.6. HRMS (ESI, TOF) m/z: calcd for $C_{28}H_{21}O_9^+$ $[M + H]^+$: 501.1186; found 501.1200. **FL-CO-2:** ^{1}H NMR (400 MHz, Acetone-*d*$_6$) δ 9.19 (s, 1H), 8.01 (d, *J* = 7.5 Hz, 1H), 7.80 (dt, *J* = 28.3, 7.4 Hz, 2H), 7.38 – 7.24 (m, 2H), 7.02 (d, *J* = 8.8 Hz, 1H), 6.92 (d, *J* = 8.7

Hz, 1H), 6.80 (s, 1H), 6.69 (q, J = 8.7 Hz, 2H), 6.10 – 5.97 (m, 1H), 5.43 (d, J = 17.2 Hz, 1H), 5.31 (d, J = 10.5 Hz, 1H), 4.76 (d, J = 5.6 Hz, 2H). ^{13}C {^{1}H} NMR (100 MHz, $CDCl_3$): δ 169.3, 160.4, 153.7, 153.6, 153.4, 153.0, 152.6, 136.3, 132.6, 131.0, 130.2, 130.1, 127.5, 125.6, 124.9, 119.3, 118.4, 118.1, 113.7, 111.2, 110.6, 103.4, 103.3, 82.7, 69.9. HRMS (ESI, TOF) m/z: calcd for $C_{24}H_{17}O_7^+$ $[M + H]^+$: 417.0974; found 417.0984.

Spectroscopic Studies

Stock solutions of **FL-CO-1** (5 mM), $PdCl_2$ (5 mM), and Pd $(PPh_3)_4$ (5 mM) were prepared fresh in DMSO. CORM-2 stock solution (10 mM) was prepared by dissolving 2.05 mg CORM-2 in 400 mL DMA; CORM-3 stock solution (10 mM) was prepared by dissolving 0.35 mg CORM-3 in 119 mL DI water. For the fluorescence experiments, 1482 μL PBS, 1.5 μL FL-CO-1 and 1.5 μL $PdCl_2$ were added into a cuvette, followed by 15 μL CORM-2 or CORM-3 stock solution. The cuvette was sealed with a cap and mixed by vortexing for 5 s. Then the fluorescence data were acquired.

Preparation of CO solution Spectroscopic Studies

For saturated CO solution, pure CO gas was bubbled into the PBS solution in the cuvette for 30 mins to make a CO saturated solution. As an example, 1.5 μL FL-CO-1 and 1.5 μL $PdCl_2$ were added into a cuvette with 1497 μL CO saturation PBS solution. The cuvette was sealed with a cap and mixed by vortexing for 5 s. Then the fluorescence data were acquired. When $PdCl_2$ or Pd(0) were added to the CO solution, we observed that the DMSO stock solution initially turned to black (gel-like no precipitation observed). After mixing thoroughly, the solution became clear, and the black coloration disappeared.

Gas bubbling experiment

1482 μL PBS, 1.5 μL FL-CO-1 and 1.5 μL $PdCl_2$ were added into a cuvette, followed by 15 μL CORM-2 stock solution. The cuvette was sealed with a cap and mixed by vertexing for 5 s. Then the fluorescence data were acquired. After around 1000s, the cuvette was removed from the fluorescence meter, and gas (CO or N_2) was directly bubbling into the system by a long needle for 2 mins. Then, the cuvette was sealed with a cap and the fluorescence data were acquired. For the vortex mixing, the cuvette shakes on the vortex for 2 mins.

Supporting Figures

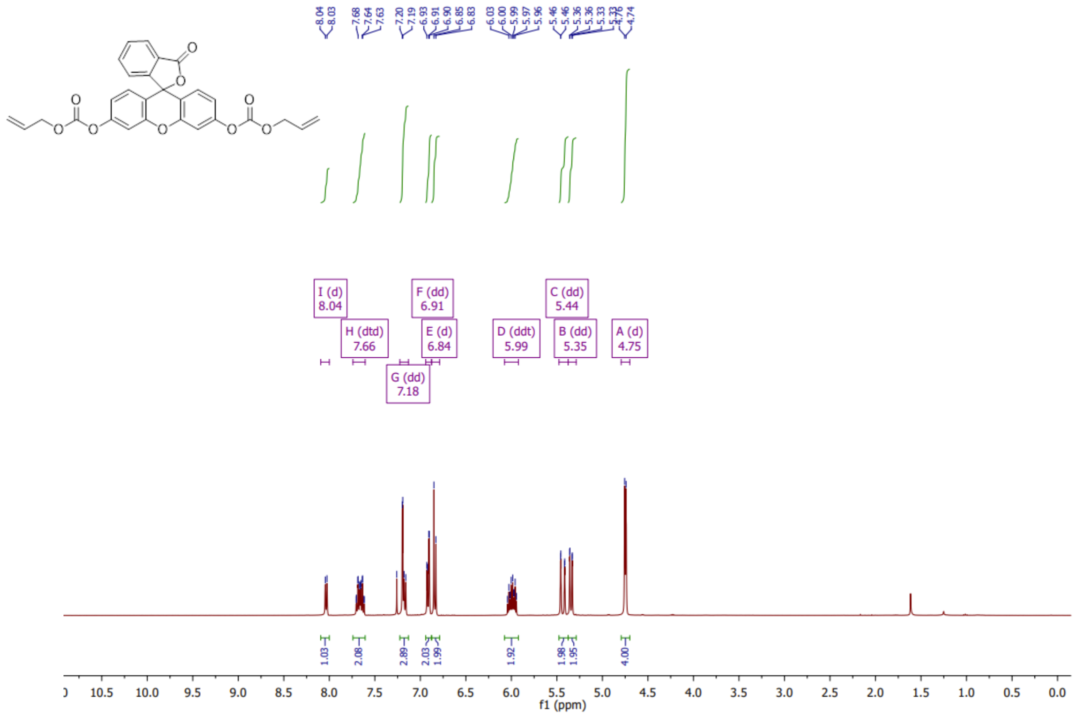

Figure S1. ^{1}H NMR (400 MHz) of FL-CO-1 in $CDCl_3$.

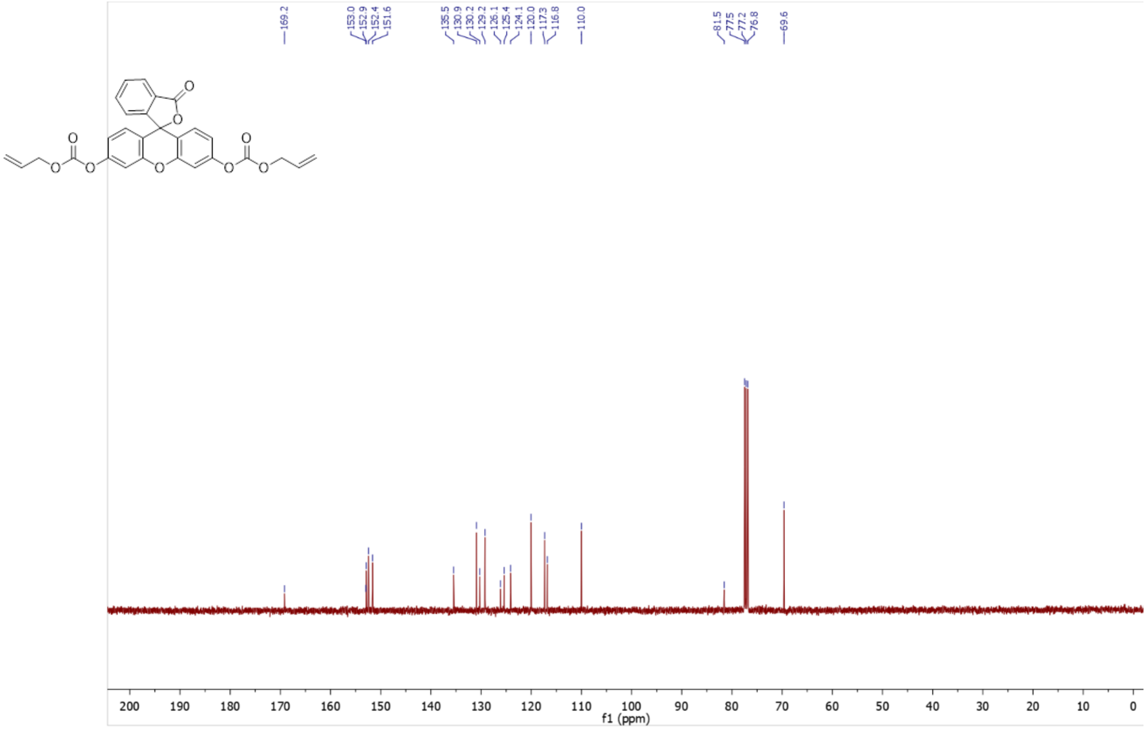

Figure S2. ^{13}C {^{1}H} NMR (100 MHz) of FL-CO-1 in $CDCl_3$.

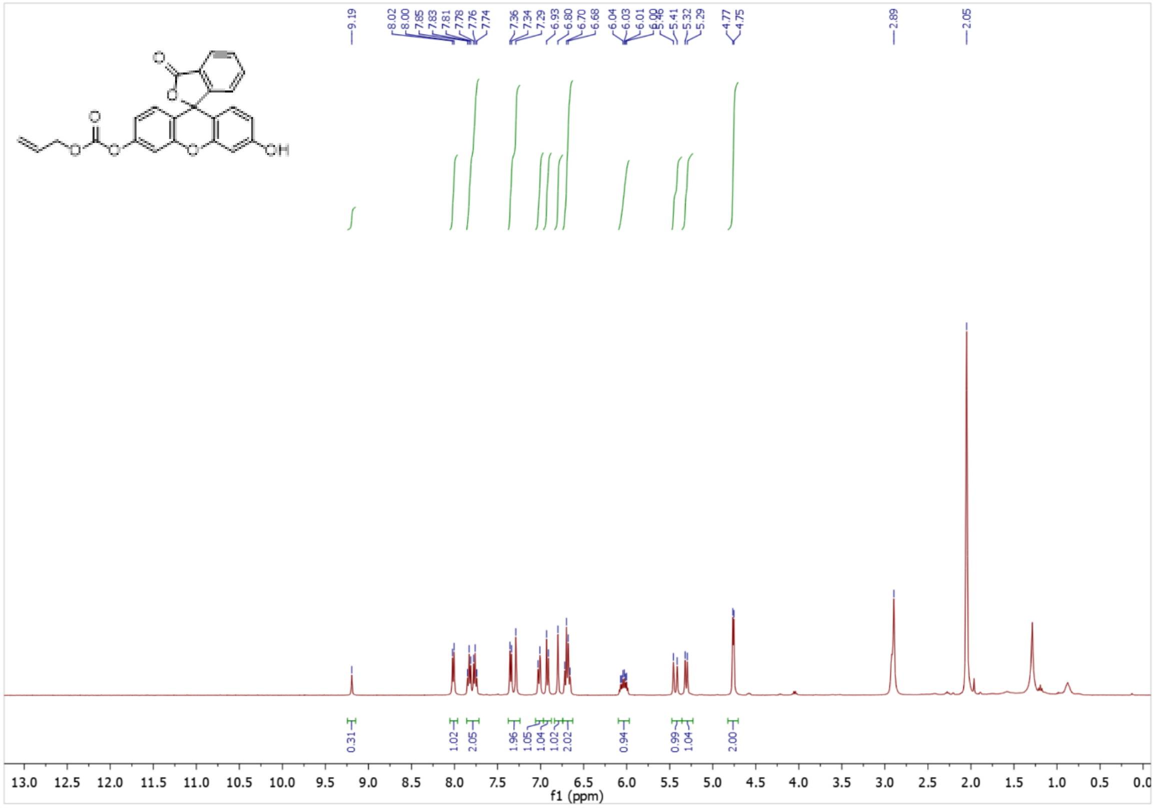

Figure S3. ^{1}H NMR (400 MHz) of FL-CO-2 in acetone-*d6*.

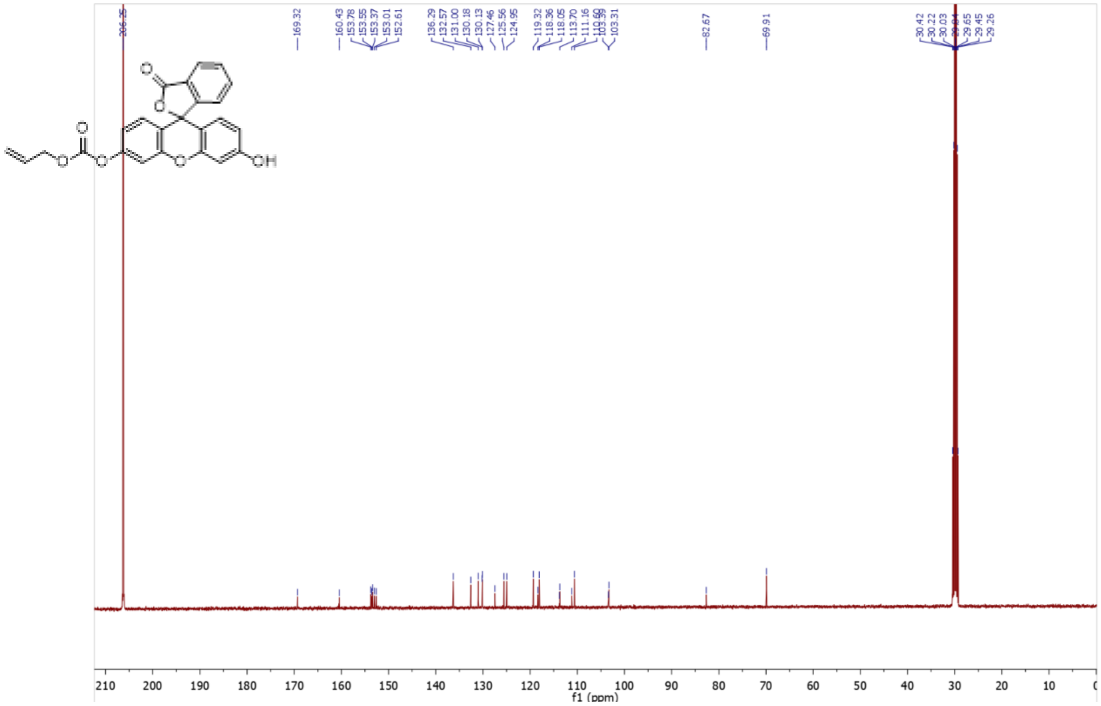

Figure S4. ^{13}C {^{1}H} NMR (100 MHz) of FL-CO-2 in acetone-*d6*.

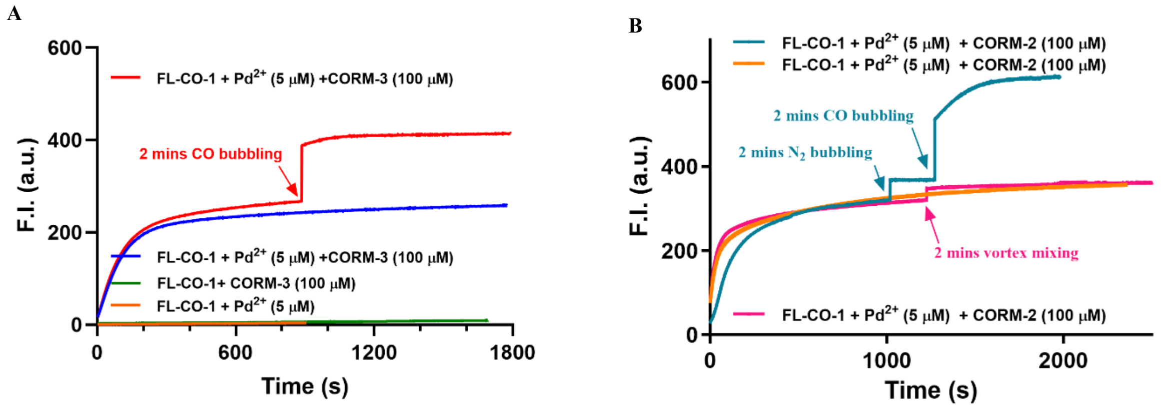

Figure S4. A. FL-CO-1 (5 μM) with CORM-3 in the present of $PdCl_2$, with/without CO gas for 2 mins. B. FL-CO-1 (5 μM) with CORM-2 in the present of $PdCl_2$, with/without CO gas and N_2 gas bubbling, or vortex mixing for 2 mins. The experiments were carried out in a mixed solution of PBS (99%), DMSO (0.2%), and DMA (0.8%) at room temperature. The cuvette was sealed with a cap and mixed by vortexing for 5 s and then kept still for the measurement. (bandwidth = 3 nm, λ_{ex} = 490 nm, λ_{em} = 515 nm)

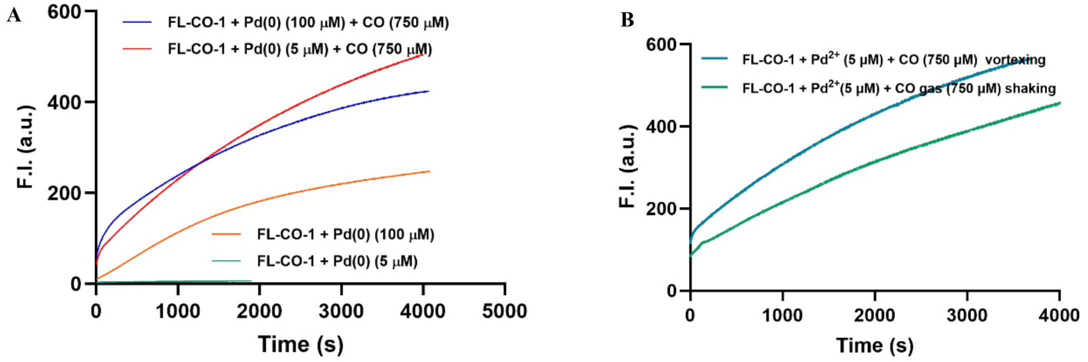

Figure S5. A. FL-CO-1 with Pd $(PPh_3)_4$ (5 μM or 100 μM) in the CO solution. B. FL-CO-1 with $PdCl_2$ (5 μM) in the CO solution, mixed with hand shaking or vortex shaking. The experiments were carried out in a mixed solution of PBS (99.8%) and DMSO (0.2%) at room temperature. The cuvette was sealed with a cap and mixed by vortexing for 5 s and then kept still for the measurement. (bandwidth = 3 nm, λ_{ex} = 490 nm, λ_{em} = 515 nm)

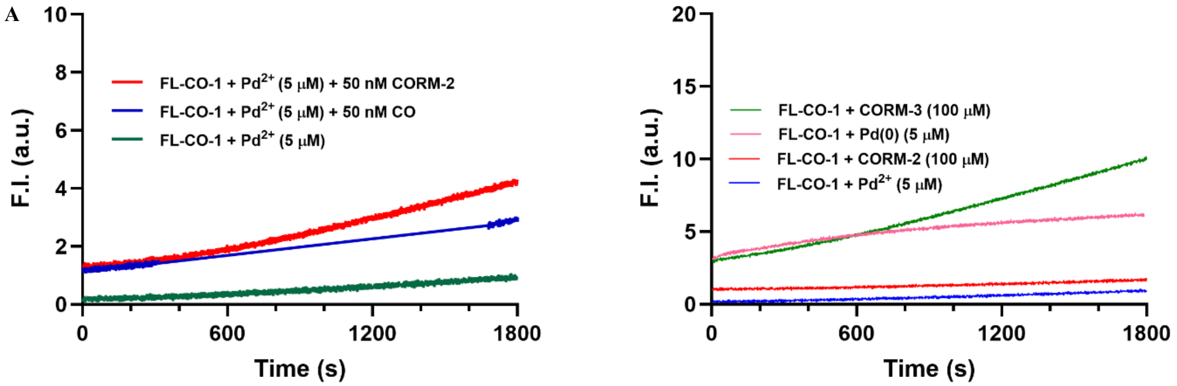

Figure S6. A. Fluorescence time-course analysis of the FL-CO-1 (5 μM) + $PdCl_2$ (1 eq) system in the present of 50 nM CORM-2 and CO gas. B. Different controls mentioned in this manuscript. The experiments were carried out in a mixed solution of PBS (99%), DMSO (0.2%), and DMA (0.8%) at room temperature. The cuvette was sealed with a cap and mixed by vortexing for 5 s and then kept still for the measurement. (bandwidth = 3 nm, λ_{ex} = 490 nm, λ_{em} = 515 nm)

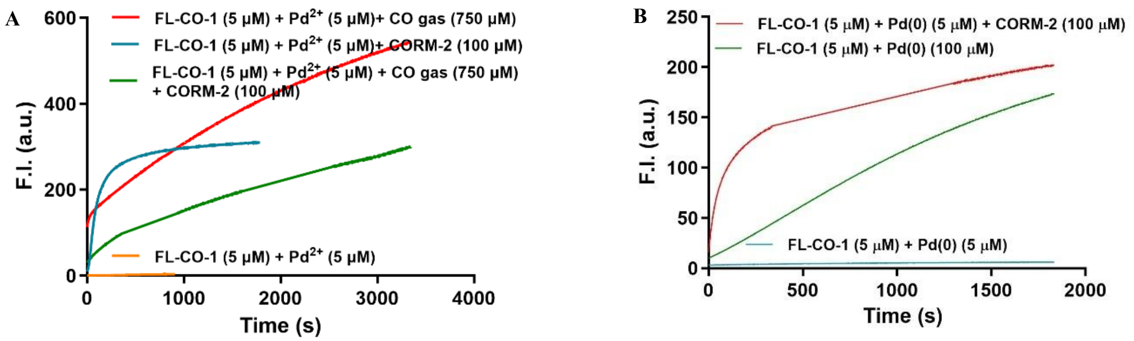

Figure S7. (A) Fluorescence time-course analysis of the FL-CO-1 (5 μM) + $PdCl_2$ (1 eq) system in the presence of CORM-2 and CO gas. (B) Fluorescence time-course analysis of the FL-CO-1 (5 μM) + $Pd(TPP)_4$ (1 eq) system in the presence of CORM-2. The experiments were carried out in a mixed solution of PBS (99%), DMSO (0.2%), and DMA (0.8%) at room temperature. The cuvette was sealed with a cap and mixed by vortexing for 5 s and then kept still for the measurement. (bandwidth = 3 nm, λ_{ex} = 490 nm, λ_{em} = 515 nm)

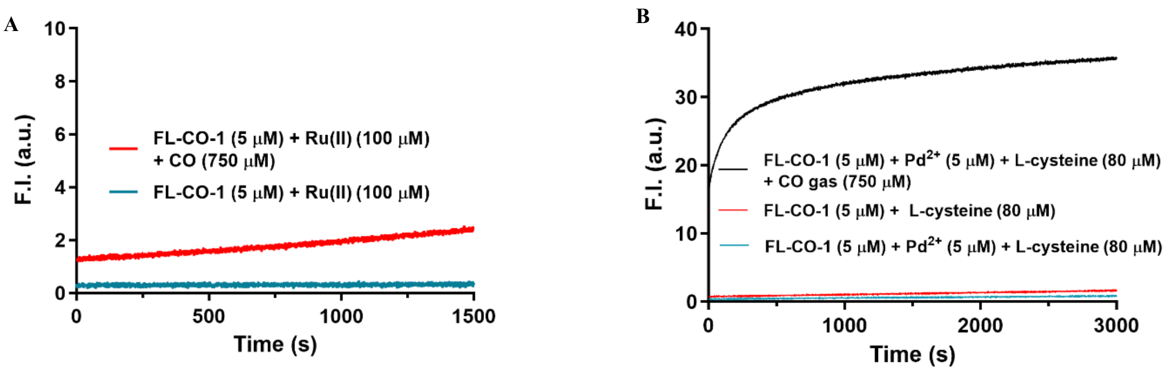

Figure S8. (A) Fluorescence time-course analysis of the FL-CO-1 (5 μM) + $Ru(bpy)_3(PF_6)_2$ (1 eq) system in the presence of CO gas. (B) Fluorescence time-course analysis of the FL-CO-1 (5 μM) + $PdCl_2$ (1 eq) system in the presence of L-cysteine and CO gas. The experiments were carried out in a mixed solution of PBS (99%), and DMSO (1%) at room temperature. The cuvette was sealed with a cap and mixed by vortexing for 5 s and then kept still for the measurement. (bandwidth = 3 nm, λ_{ex} = 490 nm, λ_{em} = 515 nm)

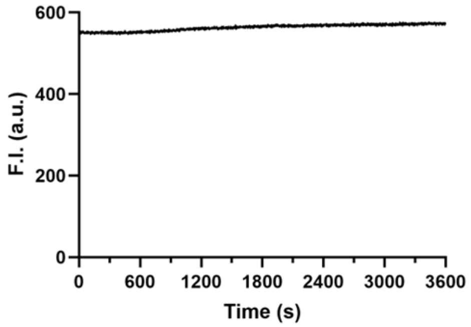

Figure S9. Fluorescence time-course analysis of fluorescein (5 μM) for 1 hour. The experiments were carried out in a mixed solution of PBS (99.8%), and DMSO (0.2%) at room temperature. The cuvette was sealed with a cap and mixed by vortexing for 5 s and then kept still for the measurement. (bandwidth = 3 nm, λ_{ex} = 490 nm, λ_{em} = 515 nm)

Reference

(1) Feng, W.; Liu, D.; Feng, S.; Feng, G. Readily Available Fluorescent Probe for Carbon Monoxide Imaging in Living Cells. *Anal Chem* **2016**, *88* (21), 10648-10653,
